# Psychometric evaluation of health related quality of life among rural-to-urban migrants in China

**DOI:** 10.1186/s12955-015-0350-1

**Published:** 2015-09-24

**Authors:** Peigang Wang, Cen Chen, Ronghua Yang, Yan Wu

**Affiliations:** Wuhan University, School of Public Health, Global Health Institute, Wuhan, China; Renmin University of China, School of Statistics, Beijing, China; Hohai University, School of International Languages and Cultures, Nanjing, China; Wuhan University, School of Information Management, Wuhan, China

**Keywords:** SF-36, Health-related quality of life, Chinese migrants

## Abstract

**Objectives:**

Our study discusses health related quality of life (HRQOL) as measured by 36-item Short Form (SF-36) for rural-to-urban migrants in China, and assesses the validity and reliability of the SF-36 for this group.

**Methods:**

In 2012,765 rural-to-urban migrant respondents chosen by probability and the non-probability sampling methods have completed the survey in Wuhan, Mid-China. The reliability of SF-36 is analyzed by Cronbach's alpha (*α*) coefficient, split-half coefficient, theta (θ) and omega (Ω) coefficient, the validity is calculated by confirmatory factor analysis (CFA) and known-group methods.

**Result:**

Split-half reliability coefficient is 0.717. Cronbach's alpha coefficient is 0.776. Theta and omega coefficient are 0.862 and 0.903 respectively. CFA statistical analysis results are shown as follows: GFI = 0.926, Chi-Square/Df = 2.059, RMSEA = 0.037, CFI = 0.939. Physical and mental component summary (PCS/MCS) scores are tabulated by known-group variables and show a statistical significance.

**Conclusion:**

In general, SF-36 is a reliable and valid instrument for measuring HRQOL of rural-to-urban migrants in China. Furthermore, Chinese migrants have lived and worked in a hard environment, their salaries are much lower than that of the counterparts, HRQOL of this group is also a little lower and deserves much attention from society.

## Introduction

In tradition, health is often viewed as a physical state. The concept of health has been changed since 1970's [1], now as being a dynamic state of well being characterized by a physical, mental and social potential [2], and not merely the absence of disease or infirmity (International Health Conference, 1946). However, WHO definition is appreciated as that it has widened the health conception from physical dimension to the physical, mental and social dimensions [2, 3]. HRQOL is defined as an individual's satisfaction or happiness with the dimensions of life insofar as they affect or are affected by health, concerning that HRQOL is concerned primarily with those factors that fall under the purview of health care providers and health care systems [4]. To date, many instruments have been developed to evaluate the HRQOL, SF-36 is generic and probably one of the most extensively used, widely translated and tested instruments worldwide [5–7].

Based on the medical outcome study at Boston Institute of Health, SF-36 was used to evaluate general health concepts relevant across age, disease and treatment groups [8]. Physical health and mental health are two major dimensions included in SF-36 measurement [8–10], and it is often applied to health policy program evaluation, general population surveys and other diverse population analysis [11]. Furthermore, SF-36 was viewed as sensitive both for the disease person [12] and for the health of general population [13, 14]. As a multi-item scale, it includes eight health dimensions [11]: physical functioning (PF, 10 items); role limitations due to physical health (RP, 4 items); bodily pain (BP, 2 items); social functioning (SF, 2 items); general mental health (MH, 5 items); role limitations because of emotional problems (RE, 3 items); energy/fatigue (VT, 4 items); and general health perceptions (GH, 5 items). Each dimension includes 2 to 10 items, and each item is rated as a two-to-six-point Likert scale.

SF-36 scale was translated into different languages and tested in more than 40 countries in the world by the International Quality of Life Assessment (IQOLA) Project. Ren et al [15] firstly used the Chinese version of SF-36 to evaluate the psychometric property of Chinese American. However this study did not confirm the acceptability and validity for Chinese people in Asia. Lam et al [16] firstly used SF-36 to test the cross-cultural validity for Hong Kong Chinese people. Fuh et al [17] tested the reliability and validity of SF-36 in its Chinese (Taiwanese) version. Later, the Chinese version of SF-36 was tested broadly in Mainland China for diverse populations, such as disease people [18], urban construction workers [19]. Nevertheless, few studies have discussed the reliability and validity of SF-36 for general rural-to-urban migrants in Mainland China.

The quality of life of the rural-to-urban migrant population is of growing concern, as their survival and development is an important agenda for current society. These rural-to-urban migrants, who are largely young, poor, single men with less education, have little or no medical care [19], limited communication with counterparts, little or low labor protection insurance and instability of living and working. However their HRQOL has rarely been reported. When these migrants are not well-prepared for migrating, and not knowledgeable enough to protect themselves, and not under the umbrella of their social network, their living and work of migration journeys will be tough [20]. The objective of this paper is to test the psychometric properties of SF-36 for using among migrants in mainland China. The results will also contribute to the growing literatures on the reliability and validity of SF-36 in different cultures and for different population. If these evaluation meet the statistical criteria and can be confirmed, then the Mainland version of SF-36 would have the potential application to the different kinds of migrants as well as those who would migrate from their native lands to other places around the world.

## Methods

### Sample and sampling procedure

A cross-sectional study was designed and conducted in August, 2012, in Wuhan. Both the probability and the non-probability sampling methods were employed. First, three out of seven districts, namely, Qingshan district, Hongshan district, Wuchang district, were chosen as the field work spots via random sampling method. Second, purposive non-probability sampling method was adopted to decide the specific migrant employment places in which rural-to-urban migrants take a relatively big part, such as hotels and construction sites. Each participant was asked to finish the questionnaire separately under the supervision of the interviewer. 842 questionnaires were distributed in the three districts and 765 copies were collected at last. Variables were input via EpiData3.1 by double-input method to ensure the quality.

Ethical approval for this study was obtained from School of Public Health Review Board of University of Wuhan prior to the implementation of the study, and oral informed consent was obtained from the participants.

### Variables

The Mainland version of SF-36 was translated from the IQOLA SF-36 Standard UK Version 1.0 by Zhejiang University, China. The Reliability and validity of the Chinese version for general population have been confirmed in Hangzhou [1, 21]. The Physical Component Summary (PCS) and the Mental Component Summary (MCS) are the two main parts of SF-36 scale. It is clustered into the following eight scales: PF (the extent to which health limits physical activities), RP (the extent to which physical health interferes with work or other daily activities), BP (the intensity of pain and the effect of pain on normal work), GH (personal evaluations of current health, health outlook and resistance to illness), VT (feeling full of energy rather than tired and worn out), SF (the extent to which physical health or emotional problems interfere with normal social activities), RE (the extent to which emotional problems interfere with work or daily activities) and MH (general mental health including depression, anxiety et al.) [22, 23]. The first four dimensions fall into PCS part, the rest four MCS part. A detailed description of the conceptual background, development, and testing of the SF-36 is available elsewhere [8, 11, 24]. In this study, each SF-36 item is coded, summed, and converted to a scale of 0–100 with 0 and 100 corresponding to worst and best HRQOL respectively [25].

### Statistical analysis

First, Split-half reliability method is employed. Split-half reliability which is often used to test the internal consistency is computed by correlating the scores of the odd half with those of the even half in each item of SF-36. In general, the split-half reliability coefficients greater than 0.7, indicate a good internal consistency. Further, we also provide the Cronbach's alpha (*α*) coefficient, theta (*θ*) and omega (Ω) coefficients to evaluate the internal consistency of the SF-36. A coefficient value of 0.7 or higher is generally considered to be sufficient to demonstrate the accepted internal consistency [8, 26]. However Cronbach's *alpha* coefficient will underestimate the reliability of SF-36 because this scale has eight domains which showed somewhat heterogeneity [27, 28]. Therefore, theta and omega coefficients are used to get more accurate estimation for the reliability. The detailed formals are as follows:

$$ \begin{array}{l}\theta =\frac{N}{N-1}\;\left(1-\frac{1}{\lambda}\right)\\ {}\\ {}\Omega =1-\frac{N-{\displaystyle \sum {h}_i^2}}{N+2r}=1-\left(1-\alpha \frac{N-1}{N}\right)\;\left(1-\frac{{\displaystyle \sum {h}_i^2}}{N}\right)\end{array} $$

“Known-group” validity is one kind of construct validity evaluation methods, which is demonstrated when the SF-36 dimensions can discriminate between two or more known subgroups to differ on the PCS or MCS scores. Known-group analysis showed good discriminate validity between socio-demographic variables with differing health states [29]. Specially, it was expected that female, widowed or divorced, older person, owning less adequate income, less education experience, or unemployment person would report much worse PCS and MCS scores [7, 29]. As for the age, increasing age would be associated with lower PCS scores and higher MCS scores [29].

Confirmatory Factor Analysis (CFA) is another important evaluation method to test the construct validity. Estimation of the best-fitting model is performed by a maximum likelihood method by the Structure Equation Model. Goodness-of-fit of the models is assessed by Chi-square/df, goodness of fit index (GFI), Root Mean Square Error of Approximation (RMSEA) and comparative fit index (CFI) indicators. Goodness-of-fit is implied with a Chi-square/df lower than 3.00, value of RMSEA less than 0.05, other values greater than 0.90. Statistical Analysis System (SAS) 9.1.3 are used for analyzing the survey data. The difference is regarded as statistically significant if the *P* value is less than 0.05.

## Results

### Sample characteristic

In the migrant sample, the proportion of female is much higher than that of male, with an average age of 29 years old currently. The vast majority of the respondents have got junior high or high school education degree, 15.82 % of the respondents had received college education degree or above. Monthly income of the vast majority of the respondents ranged from 1500 to 2000 yuan RMB, only that of 14.77 % of the respondents had overwhelmed 3,000 yuan RMB. Half of the respondents were at single status, nearly half of the respondents married. 39.14 % were in the service sectors, 11.91 % were construction workers, managers accounted for 11.91 %, a relatively small proportion of production workers, professional and technical people.

### Data quality

The descriptive statistics for the SF-36 items and summary scores were showed in Table 1. The above paragraph has introduced that each item is recoded as that higher item responses correspond to better health. Each item shows negative skewness to varying degrees except for the item “Subjective Rating of Health”, indicating the majority of respondents’ responses clustering at the upper end of the response spectrum as mainly healthy status [7, 30]. The majority of upper end distribution from PCS items overwhelmed that of MCS items, suggesting that migrants have not so much more problems interfering with work or daily activities but much more problems interfering with emotional affairs.

**Table 1:** Descriptive statistics for SF-36 items and summary scores (*N* = 765)

Items	Mean	SD	Skewness	Response frequencies (%)					
				1	2	3	4	5	6
Vigorous activities	2.28	0.65	−0.36	11	50	39			
Moderate activities	2.77	0.47	−1.93	2	18	80			
Carry the shopping	2.83	0.43	−2.55	2	13	85			
Climb several stairs	2.73	0.48	−1.44	2	24	74			
Climb one flight of stairs	2.92	0.29	−3.95	1	6	93			
Bend, kneel, stoop	2.79	0.43	−1.81	1	19	80			
Walk one kilometer and a half	2.67	0.54	−1.36	4	26	70			
Walk one kilometer	2.78	0.47	−2.10	3	17	81			
Walk one hundred meters	2.93	0.30	−4.42	1	5	94			
Bathe, dress	2.94	0.28	−4.89	1	4	95			
Cut down time	1.79	0.41	−1.41	21	79				
Accomplished less	1.75	0.44	−1.14	25	75				
Limited in kind	1.76	0.43	−1.19	24	76				
Had difficulty	1.73	0.45	−1.04	27	73				
Pain-Magnitude	4.27	0.72	−1.07	0	2	7	51	40	
Pain-Interfere	4.35	0.81	−1.54	1	2	7	39	50	
Subjective rating of health	3.31	1.06	0.22	0	28	30	25	17	
Sick easier	3.74	1.08	−0.30	3	7	38	19	34	
As healthy	3.86	0.97	−0.48	2	4	32	31	32	
Health to get worse	3.53	1.11	−0.07	4	10	44	14	28	
Health excellent	3.80	1.02	−0.58	3	6	30	32	30	
Pep/Life	4.04	1.50	−0.57	8	10	17	13	37	14
Energy	4.28	1.16	−0.74	2	6	16	24	42	10
Worn out	4.25	1.22	−0.71	2	9	12	26	39	12
Tired	4.55	1.07	−1.05	1	5	7	25	48	14
Social-Extent	4.18	0.78	−1.26	1	2	9	53	35	0
Social-Time	4.75	1.23	−0.93	2	6	6	25	28	34
Cut down time	1.69	0.46	−0.80	31	69				
Accomplished less	1.69	0.46	−0.84	31	69				
Not careful	1.65	0.48	−0.64	35	65				
Nervous	3.87	1.47	−0.26	6	16	16	24	23	15
Down in dumps	4.52	1.24	−0.92	2	7	7	24	39	21
Peaceful	4.15	1.22	−0.66	3	8	18	22	41	8
Blue/Sad	4.54	1.07	−1.14	2	4	7	26	48	14
Happy	4.34	1.20	−0.77	3	5	16	21	42	13

The percentage of respondents at the lowest and highest response category is called “floor effects” and “ceiling effects” (respectively) in the SF-36 literatures [29]. These can be used as an indication of instrument sensitivity. Both effects should be less than 20 % to ensure that the scale is capturing the full range of potential responses [30]. From Table 1, it can be seen that there are high ceiling effects excluding the dichotomous and triple response items mostly. Some studies also reported the high ceiling effect similar with our findings [31], especially for the RP and RE items, with the dichotomous nature. In the other five or six-response items, it significantly added the response options and then reduced the occurrence of high ceiling and floor effects. In a word, although some items exhibited somewhat of ceiling effect, there are no items showing high floor effect.

### Reliability

Split-half reliability is obtained through the use of Spearman-Brown formula. The split-half reliability coefficient of the SF-36 is 0.745, which indicates a good reliability for the scale. When the split-half reliability coefficients grouped by gender, marriage, age, and income, the coefficients vary from 0.681 to 0.722, suggesting that split-half reliability of SF-36 can be accepted for studying Chinese migrants.

The internal reliability of SF-36 is assessed by the Cronbach's alpha, theta and omega coefficient. As shown in Table 2, the overall Cronbach’s alpha coefficient reaches at 0.770, which exceeds the 0.70 cutoff frequently used to judge the reliability for the scale. All the other Cronbach’s alpha coefficients grouped by gender, marriage, age, and income for the dimensions are greater than 0.70, top out at 0.805. However, the Cronbach's alpha coefficients if item deleted range from 0.668 to 0.811. It suggests that each item is the important component of SF-36 in migrant people. However Cronbach's alpha coefficient estimation can’t overcome the problem of the heterogeneity between dimensions, so theta and omega coefficient are used to test the internal consistency. The theta coefficient is 0.862, the omega coefficient is 0.903. It suggests that both coefficients significantly improve the estimation level.

**Table 2:** Overall and subgroup Cronbach’s alpha coefficients of SF-36

		Cronbach's Alpha	Cronbach's Alpha if item deleted							
			PF	RP	BP	GH	VT	SF	RE	MH
Overall		0.770	0.773	0.759	0.732	0.745	0.729	0.721	0.762	0.723
Gender	Male	0.762	0.763	0.752	0.719	0.735	0.717	0.710	0.756	0.721
	Female	0.778	0.782	0.766	0.743	0.754	0.741	0.730	0.768	0.727
Marriage	Y	0.765	0.769	0.754	0.725	0.739	0.731	0.707	0.756	0.718
	N	0.783	0.787	0.773	0.747	0.751	0.737	0.745	0.777	0.739
Age	Before 80’s	0.763	0.773	0.752	0.729	0.741	0.718	0.700	0.752	0.720
	After 80’s	0.778	0.779	0.768	0.739	0.750	0.739	0.738	0.770	0.732
Settled	Y	0.795	0.791	0.787	0.772	0.763	0.764	0.761	0.785	0.747
	N	0.765	0.770	0.754	0.725	0.742	0.723	0.714	0.758	0.719
Income (RMB, Yuan)	1000-2000	0.753	0.756	0.743	0.717	0.720	0.709	0.709	0.747	0.701
	2000-3000	0.805	0.811	0.797	0.762	0.795	0.768	0.749	0.799	0.768
	Over 3000	0.733	0.741	0.724	0.705	0.696	0.685	0.668	0.720	0.687

### Validity

We can test construct validity by examining the variation in PCS and MCS scores by known-group method. Table 3 shows that the scores demonstrate expected relationships, with many socio-demographic differences being statistically significant (*p*<0.05). Male’s both PCS and MCS scores are higher than female’s (*P*>0.05), which shows the same finding with that of former studies [7], whereas some of the female’s mental health items are often found to be lower [32]. Married participants report better MCS scores than unmarried ones. We also find the expected decline in PCS scores and expected increase in MCS scores as age increasing, which is consistent with the prior findings [33–35]. Both PCS and MCS scores increasing as monthly income become more adequate in meeting basic needs. MCS scores will increase with the working years growing. Both PCS and MCS scores have no statistical significance within the different education groups. These results suggest that SF-36 dimensions have the good discriminate validity in migrants.

**Table 3:** Comparison of PCS-36 and MCS-36 scores with subgroup

	PCS			MCS	
	*n*	Mean	p value	Mean	*p* value
*Gender*					
Male	345	77.88	0.25	69.52	0.423
Female	420	76.79		68.59	
*Marriage*					
Married	367	77.80	0.76	71.93	*p* <0.001
Unmarried	383	76.79		66.21	
*Age*					
90’s	324	76.60	0.05	65.63	*p* <0.001
80’s	216	79.10		71.38	
Before 80’s	225	76.50		71.60	
*Education*					
Unschooled	16	79.00	0.14	71.45	0.58
Primary school	45	74.38		68.85	
Junior school	367	76.69		68.75	
Senior school	216	77.51		70.20	
College and above	121	79.48		67.41	
*Working years*					
1-5	379	76.57	0.41	66.64	*p* <0.001
5-10	214	78.44		70.59	
10-15	95	77.51		74.12	
Above15	77	77.26		70.01	
*Income (RMB, Yuan)*					
Below 1000	27	69.54	*p* <0.001	63.67	0.002
1000-1500	152	75.98		67.17	
1500-2000	305	77.62		68.15	
2000-2500	92	74.15		68.87	
2500-3000	76	80.21		74.74	
3000 above	113	80.54		71.35	

**Fig. 1:**
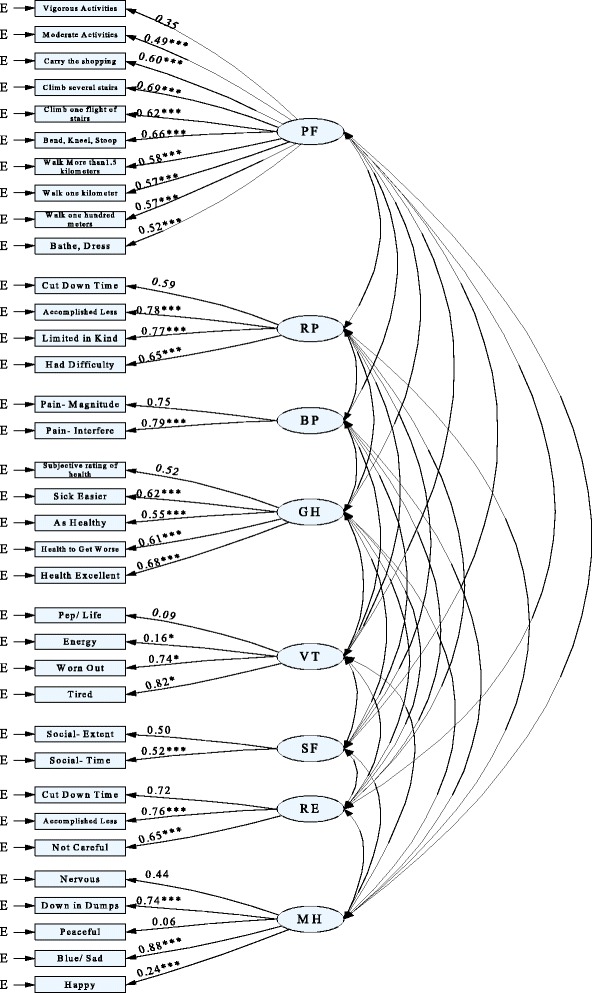
CFA analysis for the construct validity of SF-36 items. Note: ***, *p* < 0. 001; **, *p* < 0. 01; *, *p* < 0. 05

Confirmatory factor analysis (CFA) is used to test construct validity. To better understanding the construct validity of SF-36 for migrant population, the CFA results are presented in Fig. 1, the goodness of fitting indicators are: GFI = 0.926, Chi-Square/Df = 2.059, RMSEA = 0.037, CFI = 0.939. The results demonstrate that the proposed model and the actual observed data fit well. Standardized estimation for the adjusted model of the SF-36 is depicted in Fig. 1. Except the loading factor of “Peaceful” item, others are statistically significant. “Blue/Sad" has strongest effect on MH dimension, with standardized coefficients 0.88. The effect of “Peaceful" to MH dimension is the poorest, with standardized coefficient 0.06. Most items are highly loaded at the corresponding dimension. The convergent validity of the most dimensions is beyond 0.40, arriving at the judging criteria.

**Fig. 2:**
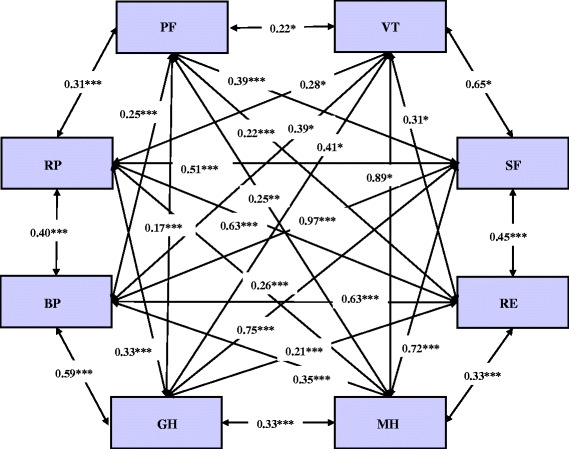
Eight latent variables of SF-36 relationship metric. Note: ***, *p* < 0. 001; **, *p* < 0. 01; *, *p* < 0. 05

Because it is difficult to exhibit the relationship for the latent variables in the Fig. 1, so we furthermore present relationship among them in Fig. 2. In the left side of Fig. 2, they are PCS dimensions. MCS dimensions are located in the right side. The results show that correlations among the PCS and MCS dimensions inside are mostly higher than those among the PCS and MCS dimensions outside. It is evident that BP with GH is the most strongly related in the PCS part. MH with VT is the most strongly related in the MCS part. BP with SF is the most strongly related across the PCS and MCS parts. These also confirm that the SF-36 has a good discriminate validity.

## Discussion

As of 2014 China’s rural-to-urban migrants have increased to 250 million since 1979. The volume of rural-to-urban migration in such a short period is likely the largest in human history [36]. Poor living conditions and inattention to health may make migrants vulnerable to poor long-term health [37]. But the studies and society have not paid so much attention to HRQOL of this group. We hope this paper can introduce one of the most useful instruments to measure the HRQOL of migrants. Based on the relatively large sampling size, the results of this paper can be extrapolated to migrants of other places in Mainland China. Empirical study confirmed that the SF-36 was valid and reliable scale for measuring HRQOL of the migrants. All items are negatively skewed as expected for the migrants similarly with other studies. The full response spectrum is used in 33 of the 35 items included in SF-36. It suggests that the scale has good sensitivity for the most items. Some highly ceiling effects can be seen for the dichotomous questions in the Table 1, also similar with the other studies (e.g., [29, 31]). All these indicators suggest that the data collected for this paper has a good quality.

As shown in the Table 2, the split-half reliability and Cronbach’s alpha for most of the SF-36 dimensions are greater than 0.70, with exception of GH, VT, SF, and MH dimensions for income subgroup, whose coefficients are slightly lower than 0.70. It suggests that migrants have a much consistency judgment for their mental health with monthly income 2000–3000 yuan group, but a big divergent reporting for their mental health beyond monthly income 3000 yuan group. When the material life arrived at certain level, people will pursuit a high level of spiritual life. At this process, some migrants will satisfy their spiritual life, some will not. So it will reduce the internal reliability in this area. In particular, SF dimensions show the lowest internal consistency reliability by Cronbach’s alpha coefficient and the lower split-half reliability. It was consistent with other studies using the SF-36 [1, 38–40] which holds that there might be some problems in the conceptualization of social function. On the other hand, understanding the difference between “what extent” and “how much of the time” and the misunderstanding of the meaning of “social activities” may lead to a low reliability for SF dimensions [39].

This survey also explores the construct validity of SF-36 for migrants. CFA is usually used to test the construct validity of an instrument. The fit results show that both GFI and CFI greatly exceed 0.90, and RMSEA is lower than 0.05, the Chi-square/df is lower than 3.00 as well. It suggests that the modified model is arrived at judge criteria of good construct validity. The standardized factor loadings from CFA model are similar with the prior studies [24, 41]. In addition, we find that among the items of SF-36, each item is strongly loaded with the corresponding dimension, with correlation coefficients varying from 0.35 to 0.69 for PF dimension, 0.59 to 0.78 for RP dimension, 0.75 to 0.79 for BP dimension, 0.52 to 0.68 for GH dimension, 0.09 to 0.82 for VT dimension, 0.50 to 0.52 for SF dimension, 0.65 to 0.76 for RE dimension and 0.06 to 0.88 for MH dimension, respectively. The lowest correlation coefficient is observed in dimension MH relating to item “peaceful”, likewise the strongest correlation comes to the item “blue/sad” in MH dimension. The scores of convergent validity for most dimension are beyond 0.40. It suggests that the SF-36 has good construct validity.

It finds that RP is closely related with RE (r = 0.63, *p* <.001). This result is consistent with the findings from other studies. There are some reasons for this high association. Rural-to-urban migrants often experience both physical and mental problems, so it is difficult for them to distinguish their physical problems from emotional ones. Former analysis showed that RP and RE were highly correlated dimensions since RE and RP dimensions contain very similar items, and these items were two-point response question [39]. This can be explained by the majority participants with high percentages in the extreme values of the scale (ceiling effect), which is recommended to include much more response categories rather than dichotomous response [8, 41]. Furthermore, both physical and mental problems interfering with each other in migrants might also be key factor to the correlation between RE and RP dimensions.

For the known-group validity, PCS and MCS scores are able to distinguish the majority of known group as the expected manner, with many socio-demographic differences being statistically significant. Faildea et al [41] finds that physical aspects are much more sensitive in detecting differences of risk factors than mental ones, but this paper gets one conclusion that mental aspects are much more sensitive. For the previous studies, it was expected that PCS and MCS scores would be lower in less educational attainment group [29, 42]. This paper found that PCS and MCS scores in this subgroup did not have significant differences. It might be due to the same lower education level for the majority of people. Previous studies reported that female had worse HRQOL than that of male. This paper also had the same findings, but it did not show significance with PCS and MCS scores. It suggested that both female and male had same physical and mental problems interfering with their works and lives.

There are some limitations in this study. First, all of the subjects are from Wuhan, one of the biggest cities in central China, but no focus has been put on the Eastern coast of China where it also has many rural-to-urban migrants for its more developed economics. So it will influence extrapolate the conclusion to other migrants. In order to get more HRQOL information of this population, further studies are needed, focusing on larger sample size from different cities, comparing scores with social change and different counterparts. Second, this paper uses the CFA model by SAS to test the construct validity instead of the more advanced estimation method- Exploratory Structural Equation Model (ESEM), because this model can improve fitting effect to obtain better results. In the future, the ESEM method would be applied as the first choice in our studies.

## Conclusion

The current study evaluates the reliability and validity of the Chinese-version of SF-36 for the rural-to-urban migrants in China. The split-half, Cronbach’s alpha, theta and omega coefficients have confirmed the developer’s claim of internal consistency for the SF-36 questionnaire. The SF-36 is valid, reliable, concise generic instrument for HRQOL of rural-to-urban migrants. Especially, theta and omega can improve the estimation effect for the reliability without considering the heterogeneity of the questionnaire. The known-group and CFA both have confirmed the good validity of SF-36 for Chinese migrants. The results of item-internal consistency are similar to the previous studies. For many reasons, the HRQOL of Chinese migrants is not so much higher than the counterparts. So society should take the intervention measures to improve their quality of life.
